# More than just co-workers: Presence of humanoid robot co-worker influences human performance

**DOI:** 10.1371/journal.pone.0206698

**Published:** 2018-11-08

**Authors:** Ashesh Vasalya, Gowrishankar Ganesh, Abderrahmane Kheddar

**Affiliations:** 1 CNRS-AIST JRL (Joint Robotics Laboratory) UMI3218/RL, Tsukuba, Japan; 2 CNRS-University of Montpellier LIRMM, Interactive Digital Human, Montpellier, France; Tokai University, JAPAN

## Abstract

Does the presence of a robot co-worker influence the performance of humans around it? Studies of motor contagions during human-robot interactions have examined either how the observation of a robot affects a human’s movement velocity, or how it affects the human’s movement variance, but never both together. Performance however, has to be measured considering both task speed (or frequency) as well as task accuracy. Here we examine an empirical repetitive industrial task in which a human participant and a humanoid robot work near each other. We systematically varied the robot behavior, and observed whether and how the performance of a human participant is affected by the presence of the robot. To investigate the effect of physical form, we added conditions where the robot co-worker torso and head were covered, and only the moving arm was visible to the human participants. Finally, we compared these behaviors with a human co-worker, and examined how the observed behavioral affects scale with experience of robots. Our results show that human task frequency, but not task accuracy, is affected by the observation of a humanoid robot co-worker, provided the robot’s head and torso are visible.

## Introduction

Robotics is now increasingly shifting to service and application fields, where robots need to collaborate with, and work in close proximity to, human co-workers. In these scenarios, it is of prime importance to understand how the presence of a robot co-worker influences the performance of humans around them. This understanding is essential not just in regard to productivity, but also in order to monitor and control any emotional and motor effects the presence of robot co-workers may have on the humans.

Observation of actions performed by others is known to induce implicit effects on an individual’s action. These effects, that are referred to as motor contagions, have been extensively studied in psychology and sports science [1–10]. In comparison, studies of motor contagions during human-robot interactions [11] are sparse, and have examined either how the observation of robots affect a human’s movement velocity [12–15], or how it affects a human’s movement variance [16–19]. However, the studies that reported changes in movement variance utilize arguably abstract tasks, and the studies reporting changes in movement speed do not analyze at how the participant movement variance changed with the speed. On the other hand, most industrial tasks require specific precisions in the movements and therefore, the performance in these tasks needs to be defined by considering both task speed (or frequency) and task accuracy. Here primarily, we analyzed how looking at a robot affects both, the speed and variance of the observing human’s movement, to see whether we can quantify how human *performance* is affected by motor contagions. Furthermore, while there is contradictory evidence to suggest that the physical form of a robot co-worker (specifically whether it is humanoid or not) does [20] or does not [17] affect the variance of movements by human’s, it is unclear whether this is also true for the case of movements speeds, and hence performance. Finally, it is unclear whether and how the performance effects due to a robot co-worker are modulated by a human co-worker’s prior experience with robots, an issue that is crucial to understand how the human performance will change with continued exposure to a robot co-worker.

**Fig 1:**
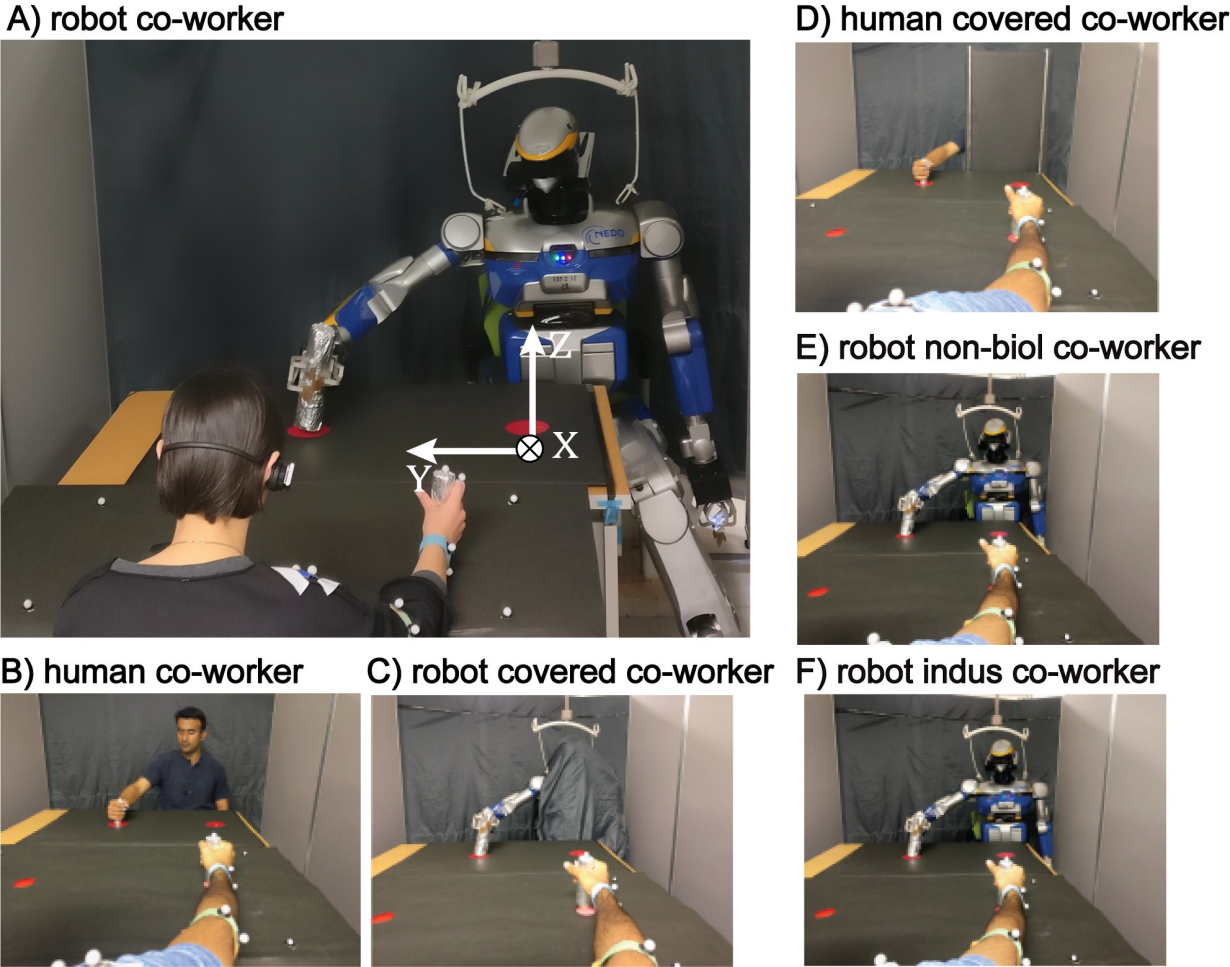
Experimental setup. The participants in our experiment worked in six conditions; with a robot performing *biological* movements in A) robot co-worker condition; B) human co-worker condition; to check relevance of human form in C) robot covered co-worker condition; and D) human covered co-worker condition; E) a robot co-worker performing *non-biological* movements in robot non-biol co-worker condition; F) a robot co-worker performing industrial movements. The coordinate axis defining the movement setup is indicated in white (A).

To address these issues, we examined an empirical repetitive industrial task in which a human participant and a humanoid robot work near each other, see Fig 1. We systematically varied the behavior, specifically frequency of robot movements and examined whether and how the frequency of movements by the human participants, and their task accuracy, is affected by the presence of the robot. To investigate the effect of physical form, we added conditions where the robot co-worker torso and head were covered, and only the moving arm was visible to the human participants. Finally, in order to compare the humanoid co-worker to a human co-worker, we also checked how the effects on the participants changed with a human co-worker, with and without his/her torso and head visible. To anticipate our results, we found that the presence of a humanoid co-worker can affect human performance, but only when it’s humanoid form is visible. Furthermore, the effect was observed to increase with prior robot experience by the humans.

## Materials and methods

### Participants

A total of 45 healthy adults participated in our study. 3 participants (2 males and a female of 3 nationalities, 29.6±5, mean±SD, aged 25-35) worked as volunteer models for the capture of human arm motion data. 42 participants (20 Males and 22 females of 12 nationalities, 25.9±4.35, mean±SD, min. age 20, max. age 39), were participated as ‘co-workers’ in our main experiment. 3 out of 45 participants were left-handed according to the *Edinburgh Handedness Inventory*, and all participants had normal or corrected to normal vision. The experiments were approved by the local ethics committee at the National Institute of Advanced Industrial Science and Technology (AIST) in Tsukuba, Japan, and all participants read and signed an informed consent form along with the PLOS consent form for the usage of their images in the paper before taking part in the experiments. Participants were well instructed and informed with the experiment and task procedure, however they were naïve to the motives (participants were not told what aspect of their behavior we were analyzing in the experiment) of the experiments to avoid bias in the results as we are interested in the implicit effect of motor contagion. Each Participant received 2021 Japanese Yen (JPY) to participate. Participants for our study were recruited through an advertisement via a local event forum, Facebook page of our experiment and via word of mouth in the Tsukuba University, Tsukuba, Japan. Participants needed to be at least 18 years old to participate in this experiment, apart from that, there were no restrictions.

### Setup

The participant and co-worker (a human or a humanoid robot) were seated on a chair with tables facing each other as shown in the experiment setup (Fig 1). On a horizontally placed touch-screen (23-inch HD DELL, P2314T) on the table, participants were presented with two red circles of diameter ⊘5 cm at distance of 50 cm from each other. The co-worker was similarly presented with two red circles on black cardboard ⊘9 cm at distance of 50 cm. The whole setup was enclosed by movable panels and the panel behind the co-worker was covered with a dark grey curtain. A motion tracking system (Motion Analysis Co.) with six infrared cameras (kestrel) and ten passive markers were used to record the arm motions of the participant and co-worker at 200Hz. A bipedal HRP-2Kai [21] humanoid robot (154 cm tall, 58 kg, 32 degrees of freedom) was used as the robot co-worker (Fig 1). A well-trained experimenter (M, 37) acted as the human co-worker. Both co-workers used their right arm throughout the experiment.

### Experimental task and conditions

Motivated by the hand movements during an industrial pick-and-place, or parts assembly task, our task required participants to repeatedly touch two static red circles on the touch-screen with a stylus in their right hand during the task (Fig 1). While the participants were asked to touch the circle in each trial, they were free to touch anywhere on the circle. In preliminary experiments, we found that the standard deviation of the participant’s touches were less than 1 cm (both in the *x* and *y* directions), and we purposely chose the radius of the touched circles to be more than 2 times larger than their standard deviation (targets were of 5 cm in diameter). This large size was crucial because, while the participants were asked to touch the circle in each trial, they were free to touch anywhere on the circle. The large target size therefore enabled us to observe any change in participant’s touch location (that may accompany contagions in their speed), in terms of position and standard deviation, across our experiment.

**Fig 2:**
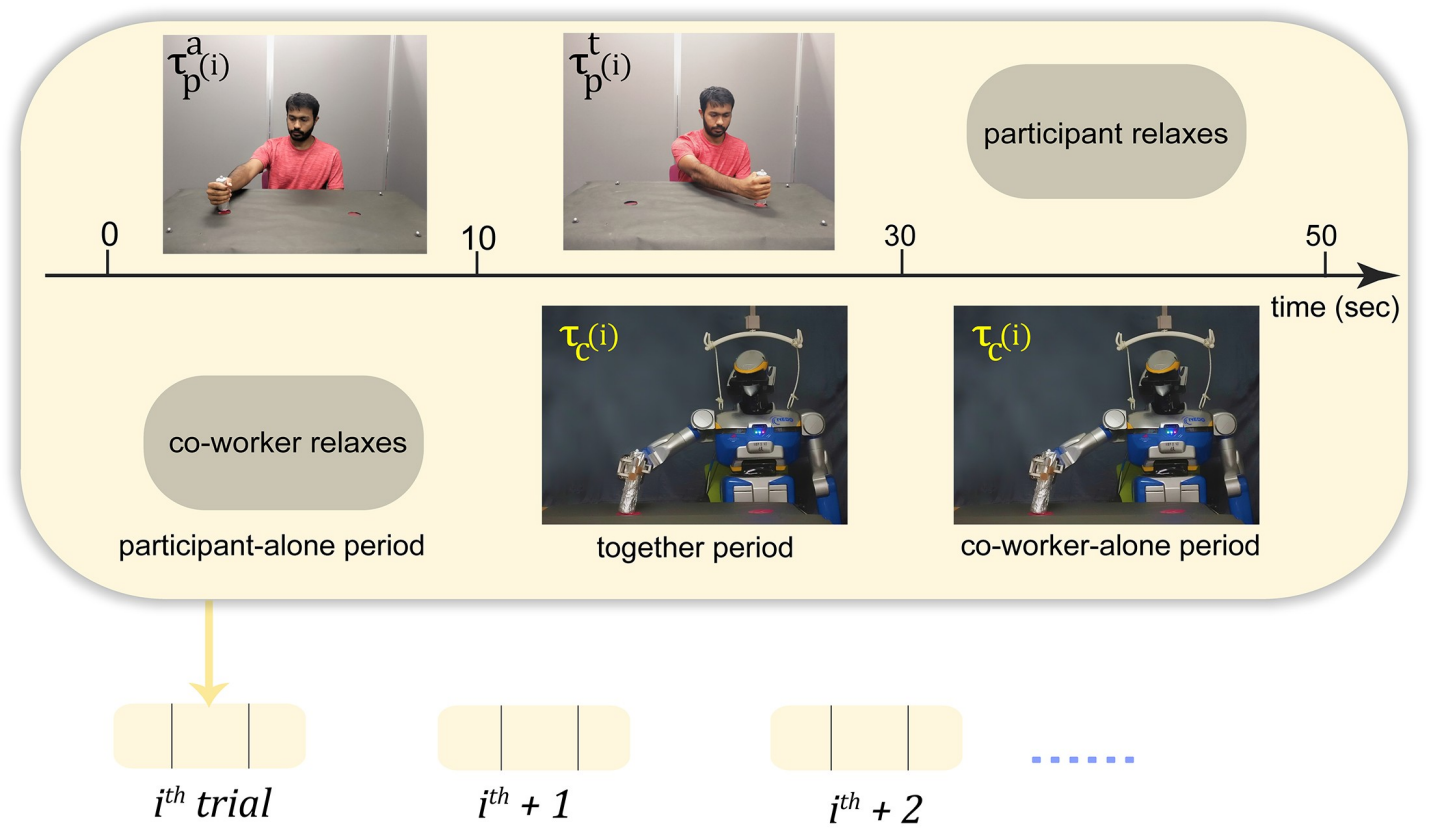
Trial protocol. The participants worked in repeated trials with either a robot or human co-worker (the figure shows the trial with a robot co-worker). Each trial consisted of period when the participant worked alone and co-worker relaxed (participant-alone period), both worked together (together period), and the co-worker worked alone (co-worker-alone period). The notation of the time variable (represented in general by *τ*) in each period are shown.

A co-worker (human or HRP-2Kai) worked on the same task in front of the participants. The participants were asked to perform their task at their own chosen ‘comfortable’ frequency, and ignore the co-worker. The participants worked in a series of 50 second trials with the co-worker. In a trial, participants initially performed alone for 10 seconds (participant-alone period), performed with the co-worker for next 20 seconds (together period) and then relaxed while watching the co-worker performs the task for the last 20 seconds (co-worker-alone period) (Fig 2).

All participants wore ear buds and headphones (through which we sent white noise) and had no external audio feedback (confirmed in the post experiment questionnaire, Q6). They were instructed to “*always hold the stylus like a stamp and touch alternatively inside each red circle on the touch-screen with continuous and smooth hand movements at a comfortable speed*”. They were specifically told to “*focus on your own task and ignore the co-worker when he/it starts after them*”. No instructions were given regarding the speed and movement trajectory.

We studied six experimental conditions. The participants worked with a HRP-2Kai humanoid robot co-worker in four conditions, specifically, a) *robot co-worker* in which the whole robot was visible to the participant, and the robot played back biological movements, b) *robot covered co-worker*, in which the robot played back biological movements, but its head and torso were covered, such that the participant could only see the robot’s moving arm. c) *robot non-biol co-worker*, in which a fully visible robot performed *non-biological* arm movements, d) *robot indus co-worker*, in which a fully visible robot performed *industrial* arm movements and they worked with a trained human experimenter in the remaining e) *human co-worker* and f) *human covered co-worker* (where the head and torso of the human experimenter were covered) conditions, (see subsection HRP-2Kai movement trajectories).

The experiment for each participant consisted of working in 3 conditions. Each participant was assigned to one of 6 *condition combination* groups, see Table 1, each with the robot co-worker condition (main), in addition to two out of five other conditions. The order of the conditions was balanced across the combination groups. This allowed us to compare the behavior of the same participants in each condition in a combination group, with their behavior in the robot co-worker condition.

**Table 1:** Condition combination groups (G). HRP-2Kai in robot co-worker (RV), robot covered co-worker (RC), robot non-biol co-worker (RN), robot indus co-worker (RI) conditions and Human experimenter in human co-worker (HV), human covered co-worker (HC) conditions. The order of conditions in a combination group were randomized across participants.

Sessions/Groups	G1	G2	G3	G4	G5	G6
Session 1	RI	RC	HV	RC	RN	RC
Session 2	RV	RI	RV	HC	HV	RN
Session 3	RN	RV	HC	RV	RV	RV

Each condition had 10 trials. The co-worker performed at a constant, pseudo-randomly selected frequency (in the range of 0.16 to 1.1 Hz) in each trial. The pseudo-random nature of the co-worker performance was critical to avoid behavioral drift contamination across trials. The human co-worker was provided with a metronome using earphones like in [15], to cue and help maintaining the movement frequency in each trial.

The robot movements in the robot co-worker conditions were a playback of the movements recorded from a previous human volunteer (see subsection HRP-2Kai movement trajectories for details). We quantified the participant performance in the trials by their half time periods or *htp* (the average time between two consecutive alternate touches, measured using motion tracking), and the variance of their press location (measured as a change of mean and standard deviation of their touch-screen presses in the X-Y plane).

### HRP-2Kai movement trajectories

**Fig 3:**
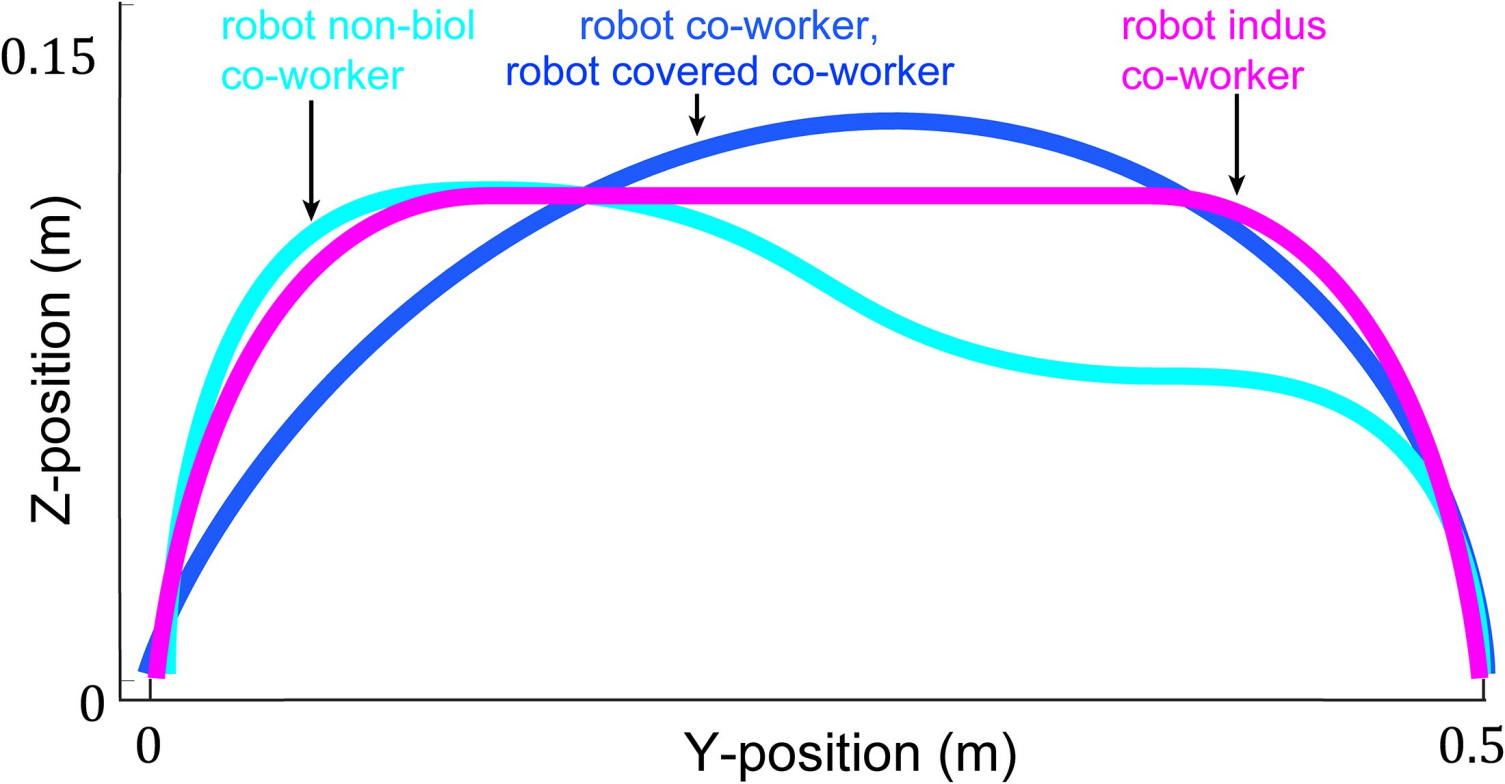
HRP-2Kai movement trajectories. The trajectories played by the robot in robot co-worker, robot covered co-worker, robot non-biol co-worker and robot indus co-worker conditions.

The biological movements played on HRP-2Kai in robot co-worker and robot covered co-worker conditions were a playback of the human arm movements (Fig 3, blue plot) recorded in a preliminary experiment with three volunteers using the same (Motion Analysis Co.) motion tracking system, while the human movements were cued by an audio metronome. Movements were collected at several frequencies between 0.16 to 1.1Hz. We found the movements of the three volunteers to be statistically similar in the Cartesian velocity profiles (p>0.05), and showing similar trend in movement height with movement frequency –trajectory height consistently decreased with increase of movement frequency. We therefore chose to use the movements recorded from one volunteer (a male) in this experiment.

**Fig 4:**
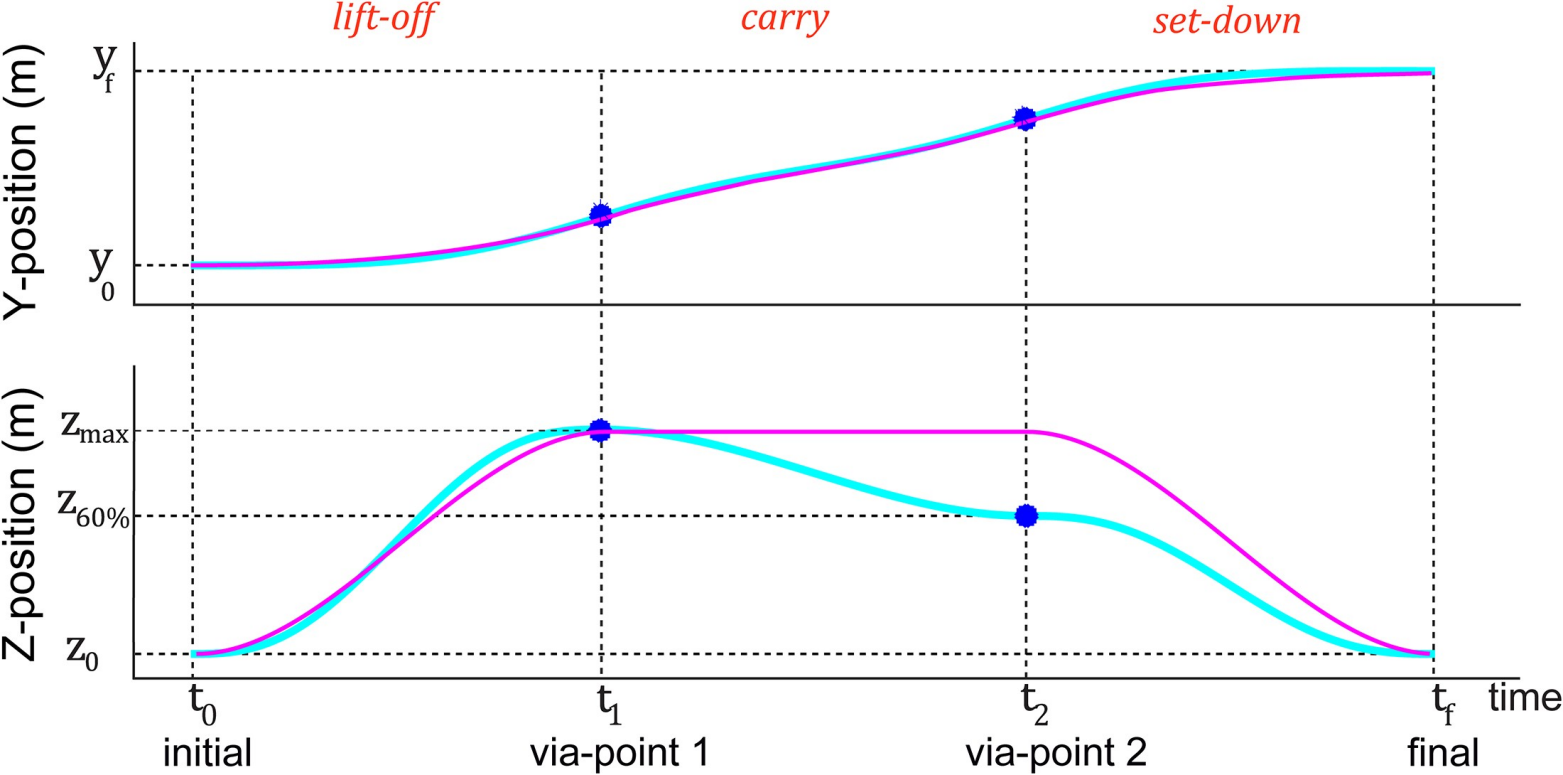
HRP-2Kai trajectory generation. The time trajectories in the Y and Z axis by the HRP-2Kai in the robot non-biol co-worker and robot indus co-worker condition, and the via-points (blue circles) used to generate both trajectories.

Well learnt human movements are characterized by a bell-shaped velocity profile. The peak of the bell-shaped profile may be shifted forward in time when precision is required at the reach end (like in our task when the participants required to touch inside a given target region), but the velocity profile is normally characterized by a single peak. Therefore, to develop a ‘non-biological’ movement profile for the robot non-biol co-worker condition, we developed a movement profile with multiple velocity peaks. This profile was developed in position-time (cyan plots in Figs 3 and 4) profile using fifth and third order polynomial segments (lift-off, carry, set-down) [22]. We observed that human volunteers’ movements to predominantly be in the Y-Z plane. The piece-wise polynomial trajectory for the robot non-biol co-worker condition was designed over the *y* (horizontal) and *z* (vertical) dimensions, while *x* was always kept constant zero.

The industrial trajectory was characterized by a constant velocity phase. Inspired from the industrial manipulators, and keeping in mind our HRP-2Kai joint constraints during fast movements, we improvised the traditionally used industrial trapezoidal velocity profile, with a third order velocity sections in the acceleration and deceleration phase (magenta plots in Figs 3 and 4). Again, since our movements were restricted in the Y-Z plane, we designed our smooth trapezoidal trajectory over the *y* (horizontal) and *z* (vertical) dimensions, while *x* is kept constant and zero. The Z elevation (*z*_max_) in this trajectory was set to 13 cm when the robot moved from left to right, and 8 cm during the return.

### Variables

Our analysis is based on the position data from both the participant’s and co-worker’s stylus markers. To extract out possible behavioral differences between the movements forward and backward, between the touch points, we analyzed behavioral variables across each movement between the red circles on the touch-screen, which we call as iterations (such that two iterations make a movement cycle). As participants and co-workers were required to make non-stop continuous movements between touches, we could extract individual iterations of participant’s and co-worker’s by looking for changes in the direction of *y*-velocity in the recorded motion capture data. In this study, we were interested in the task performance of participants, and therefore we primarily concentrated on the time between the alternate touches in each iteration, which we refer to as the half-time period (*htp*) or *τ*, and the location of their touches on the touch-screen (in the X-Y plane). In addition, we also analyzed various measures of position, velocity and acceleration along the Y (horizontal) and Z (vertical) axes over each iteration. However, these results are out of scope of this study.

### Data analysis

**Fig 5:**
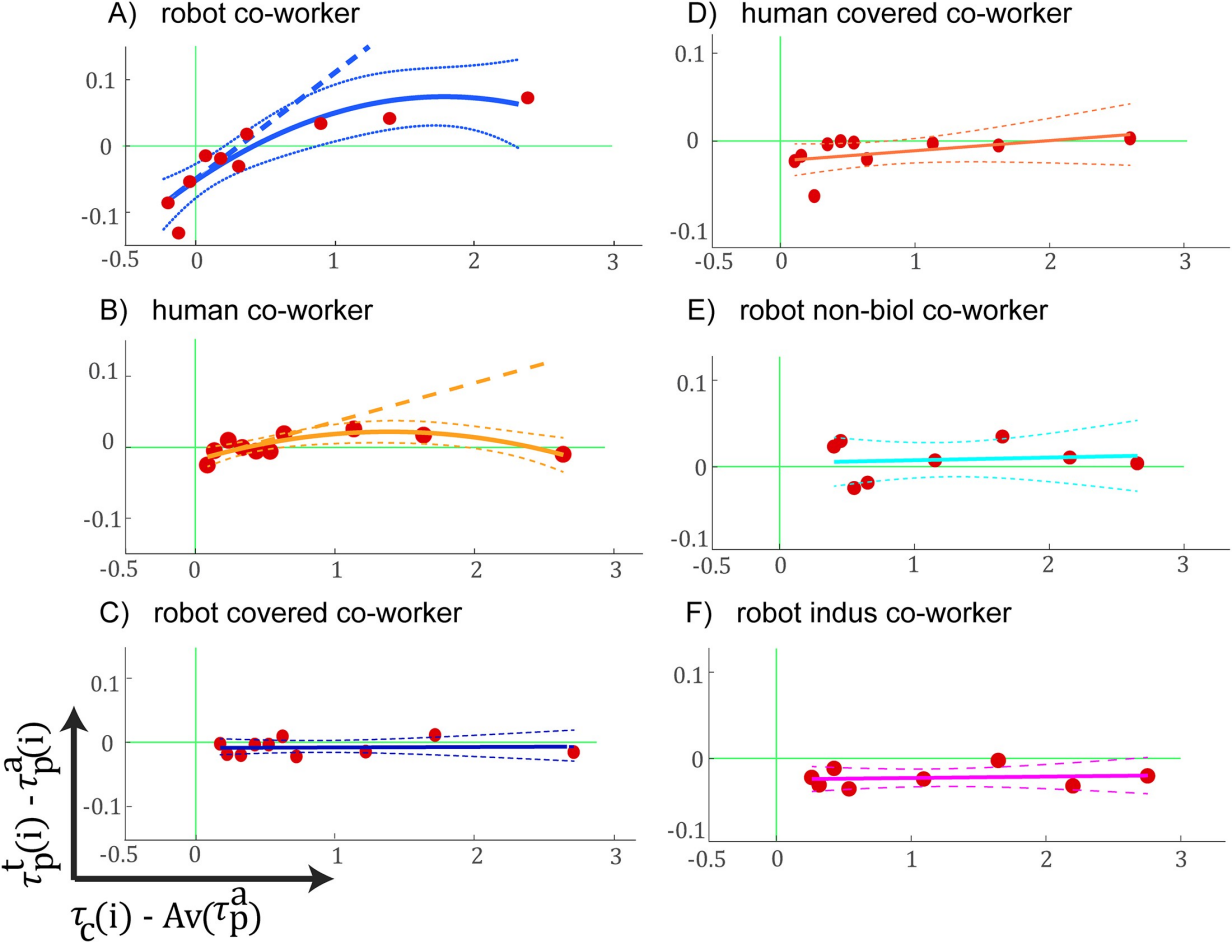
Examples of linear regression fits. The change of participant’s *htp* (the average time between two consecutive alternate touches) between the together period and alone-period ($\tau_{p}^{t}\left(i\right)-\tau_{p}^{a}\left(i\right)$), relative to the *htp* of the co-worker behavior in the same trial ($\tau_{c}\left(i\right)-Av\left(\tau_{p}^{a}\right)$), where $Av(\tau_{p}^{a})$ represents the average undisturbed *htp* by a participant across all his/her participant-alone periods. Note that the (robot or human) co-worker *htp* was random across trials, and the data in plots here are the ensemble of the participant behaviors arranged in increasing co-worker’s *htp* on the abscissa. Each plot represent a condition, A) robot co-worker (blue); B) human co-worker (orange); C) robot covered co-worker (dark blue); D) human covered co-worker (dark orange); E) robot non-biol co-worker (cyan); F) robot indus co-worker (magenta) conditions. We used the AIC to choose either a first or second order model to fit the data for each participant. The lines represent the tangent slopes at the minimal data abscissa value.

**Fig 6:**
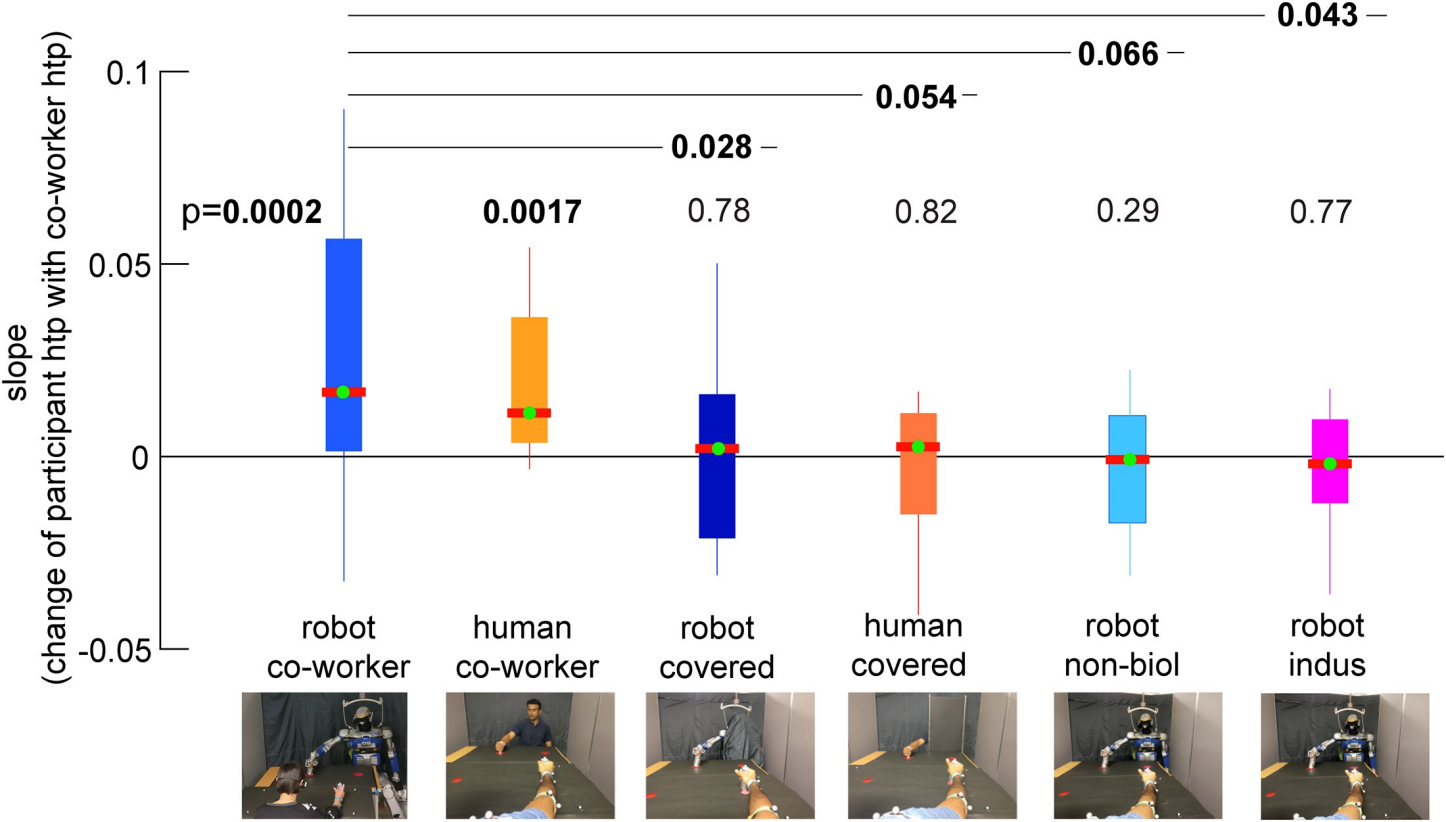
All six conditions *htp* comparision. The plot of the collection of slopes which is obtained in Fig 5 and S1 to S6 Figs supplementary figures. The condition-wise comparison of the change of participants *htp* with co-worker *htp*. P-values are Bonferroni corrected where required. The tangent slope at the minimum data abscissa value ($\min[\tau_{c}\left(i\right)-Av\left(\tau_{p}^{a}\right)]$) was collected across participants (as shown in Fig 5), checked for normality using the Shapiro-Wilk test and then analyzed for difference from zero using a one sample T-test (in case the distribution was normal) or a Signed Rank test.

**Fig 7:**
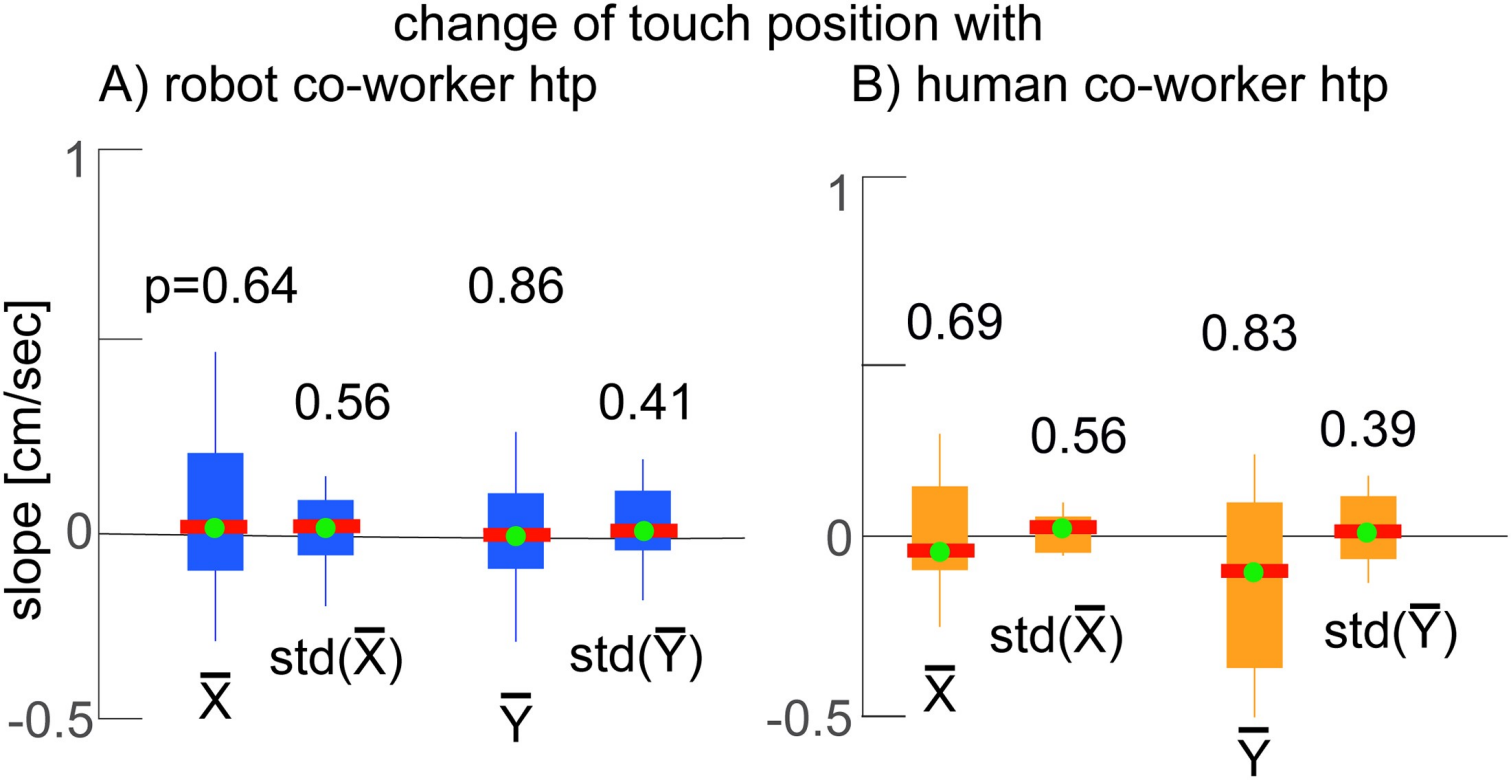
Participants touch variance. Change of participant touch position with A) robot co-worker *htp*; B) human co-worker *htp*. A similar procedure which was used to quantify *htp* was also used here (see subsection Data analysis) to analyze the change in a participant’s average X press location, average Y press location, standard deviation of X press location, and standard deviation of Y press locations relative to the *htp* of the co-worker behavior in the same trial.

We quantified the motor contagion in a participant’ *htp* (the average time between two consecutive alternate touches) by analyzing the change of participant’s *htp* between the together period and alone-period (see Fig 2) in a trial ($\tau_{p}^{t}\left(i\right)-\tau_{p}^{a}\left(i\right)$), relative to the *htp* of the co-worker behavior in the same trial ($\tau_{c}\left(i\right)-Av\left(\tau_{p}^{a}\right)$), where $Av(\tau_{p}^{a})$ represents the average undisturbed *htp* by a participants across his/her participant-alone periods. This data was regressed with either a first or second order regression model that was chosen based on the Akaike Information Criteria (AIC) [23] and by using MATLAB’s fitlm function for each participant. The tangent slope at the minimum data abscissa value ($\min[\tau_{c}\left(i\right)-Av\left(\tau_{p}^{a}\right)]$) was collected across participants, checked for normality using the Shapiro-Wilk test and then analyzed for difference from zero using a one sample T-test (in case the distribution was normal) or a Signed Rank test. The fitting of *htp* in one sample participant from each of the six reported conditions are shown in Fig 5, and the plot of the collection of slopes are in shown in Fig 6. A similar procedure was used to analyze the change in a participant’s average X press location, average Y press location, standard deviation of X press location, and standard deviation of Y press locations relative to the *htp* of the co-worker behavior in the same trial ($\tau_{c}\left(i\right)-Av\left(\tau_{p}^{a}\right)$). The slopes from these analysis are shown in Fig 7.

Next, to check the relevance of the human form, we conducted the robot covered co-worker and human covered co-worker conditions, in which the head and torso of the co-worker was covered and only the moving arm was visible to the human (see Fig 1C and 1D or inset photos in Fig 6). All other experimental settings and analysis were same as in the robot co-worker and human co-worker conditions.

### Participant sample size

As the effects of the robot co-worker condition was the focus of our experiments, each of our participant worked in the robot co-worker condition, and two of the remaining 5 conditions (human co-worker, robot covered co-worker, human covered co-worker, robot non-biol co-worker and robot indus co-worker). Note that due to the fact that each of our conditions lasted over 20 minutes, resulting in more than 1 hour of total experiment time for the three conditions, we could not have every participant participating in all the conditions. We initially recruited 35 participants to have 14 participants in each of the five conditions (giving five participant groups each of whom participated in one of the five conditions in addition to the robot co-worker condition), so as to enable a intra participant one sample T-test between the robot co-worker and each of the remaining conditions. The number ‘14’ was chosen as it corresponds to participant numbers in similar previous studies [14, 15] and corresponds to a power analysis using G* power for a 2-tailed, one sample T-test (*α* = 0.05, *β* = 0.85, *d* = 0.9) [24, 25]. However, we found that with these participant numbers, the slopes in the same robot co-worker condition were not similar among the participant groups (p<0.05, one-way ANOVA). The robot co-worker condition slopes were significantly different from zero with two participant groups (p = 0.022, and p = 0.038), tended to be significant in two (p = 0.07, and p = 0.08) and not significant in another (p = 0.36). As a majority of the values tended to be significant, we decided to increase the participant numbers by 50% (7 participants) across the participant group that were tending or not significant (these included participants who participated also in the robot covered co-worker, robot non-biol co-worker, and robot indus co-worker conditions), making a total of 42 participants. With this participant number, the robot co-worker *htp* slopes were observed to be similar across the participant groups (H(4), p = 0.99; one-way Kruskal-Wallis H-test). After removal of three outliers, this gave us participants numbers of 13 (human co-worker condition), 13 (human covered co-worker), 17 (robot covered co-worker), 17 (robot non-biol co-worker), 18 (robot indus co-worker), and 39 in total for the robot co-worker condition, see Table 2.

**Table 2:** Participant sample size.

condition	sample size
robot co-worker	39
human co-worker	13
robot covered co-worker	17
human covered co-worker	13
robot non-biol co-worker	17
robot indus co-worker	18

### Questionnaire

#### Perception and fatigue

Each of the participant in our experiment answered a short post experiment questionnaire consisting of 6 questions. The participants were asked to choose a score on a scale of 0 to 7, where 0 (Not at all), 7 (very strongly), for each of these questions, individually for every session they participated in:

Q1 My movements were affected when the agent was working with me.
Q2 My movement speed was changed when the agent was working with me.
Q3 I was tired during the experiment.
Q4 I could maintain the movement speed that I wanted even when the robot was performing its task.
Q5 I found it difficult to do my task when the agent was working with me.
Q6 I could hear noises from the co-worker during the experiment.

Q1, Q2, Q4 and Q5 were designed to access whether the participants cognitively realized the affects on their behavior due to the co-worker. A score close to one in Q1, Q2 and Q5 (and a score close to 7 in Q4) indicates that they did not consciously realize the effects. Therefore we considered the Q4 scores by subtracting the reported values from 7.

#### Robot exposure questionnaire

Following the end of our data collection, we also noted the need to measure the participant’s robot experienced and exposure to robots. We therefore sent them a questionnaire of four questions:

RQ1 How many hours do you see and/or read about robots on average per week (include robots on TV)?
RQ2 If you work with robots currently, how many hours do you work with robots (or on robotics related topics) per week?
RQ3 If you have worked with robots, but do not work anymore, how many hours have you worked on them?
RQ4 How will you rate your knowledge of robots?

For each question, the participant had to answer in hours and chose between ‘0’, ‘less than 5’, ‘5-10’ ‘10-15’, ‘15-20’, ‘20-25’, ‘25-30’, ‘more than 30’.

### Statistical correction

As reported earlier, every participant in our study participated in three conditions: the robot co-worker condition, and two of the remaining conditions. We thus compare the behavior of the participant in any condition with the robot co-worker. For each participant, there were thus two comparisons made. Correspondingly, in our comparisons in Fig 6, we use a Bonferroni correction of (3 conditions—1) 2, and all p values below 0.05 were multiplied by 2. Therefore, note that all the comparisons between conditions in Fig 6 are between equal number of participants (and we use a one sample T-test).

## Results

### Robot behavior influences human movement frequency

Fig 5 shows the change of participant’s *htp* (the average time between two consecutive alternate touches) between the together period and alone-period ($\tau_{p}^{t}\left(i\right)-\tau_{p}^{a}\left(i\right)$), relative to the *htp* of the co-worker behavior in the same trial ($\tau_{c}\left(i\right)-Av\left(\tau_{p}^{a}\right)$), where $Av(\tau_{p}^{a})$ represents the average undisturbed *htp* by a participants across all his/her participant-alone periods. Note that the (robot or human) co-worker *htp* was random across trials, and the data in Fig 5 is an ensemble of the participant behaviors arranged in increasing co-worker’s *htp* on the abscissa. We then collected the slope of the polynomial at the lowest data abscissa as a measure of how the participant *htp* was affected by the co-worker *htp*. In the robot co-worker condition the slope distribution was not normal across the participants (p<0.05, Shapiro-Wilk test, median = 0.017) and was significantly positive across participants (median = 0.017, Z(38) = 3.70, p = 0.0002, Signed Rank test). The positive slopes (light blue data in Fig 6) show that the robot performance *htp* (hence frequency) influenced the human participants. First, the human participant’s *htp* increased when the robot *htp* was longer (see first quadrants of Fig 5A), but for several participants, this increase had a threshold after which the participant’s *htp* decreased. This behavior is the reason why we found a second order fit to explain the data better with many participants using AIC. Second, the participants *htp* also decreased when the robot *htp* was shorter (3rd quadrants of Fig 5A, only across the participants in robot co-worker condition) indicating that a faster robot made the participants frequency higher. The *htp* results were similar with the human co-worker. The positive slopes (orange data in Fig 6) show that the human co-worker’s performance *htp* (hence frequency) influenced the human participants (median = 0.012, p = 0.0017, Signed Rank test).

### Press accuracy in the human not affected by robot co-worker

Studies in motor control have exhibited that human movements are constrained by motor noise, which increases with the magnitude of motor commands in the muscles [26]. In the case of ‘regular’ and automatic movements in daily life, this leads to a trade-off between the speed and accuracy of the movement [27]. However, the accuracy of movements is also modulated by the regulation of arm impedance by muscle co-contraction [28–30]. As mentioned earlier, to comment on the task performance of the human co-worker, we next analyzed whether and how the touch accuracy of the participants changed alongside the contagions in their *htp*.

Again, note that the target circles provided to the participants were large (5 cm diameter), and there were no constraint on where inside the target they touched. Therefore the participant’s touches can change (in position and/or variance) with their movement speed, without violating the task. However, interestingly we found that while the participants *htp* (hence movement frequency) changed, there were no such trend in the participants press (task) accuracy.

The mean touch positions were ($\bar{X}=0.95\;\text{cm}$; $\bar{Y}=1.17\;\text{cm}$) from the circle center across the participants, and showed a mean standard deviations of (std(X) = 0.23 cm; std(Y) = 0.79 cm) (across participants) when they worked alone (in the participant-alone period). And crucially, the participants maintained the same touch positions (there was no change of mean touch positions $\bar{X}:\text{p}=0.64$; $\bar{Y}:\text{p}=0.86$) and mean standard deviations (change of std(X): p = 0.56; std(Y): p = 0.41) between when they worked alone and when they worked with the robot co-worker (Fig 7A), showing that the robot did not affect their task accuracies.

The press accuracy was similarly constant in the human co-worker condition in which the participants worked with another (unfamiliar) human, with no observed changes in the mean touch positions ($\bar{X}:\text{p}=0.69$; $\bar{Y}:\text{p}=0.83$; Fig 7B) and mean standard deviations (std(X): p = 0.56; std(Y): p = 0.39; Fig 7B). Here note again, that the touched circles were relatively large (5 cm) and the participants could have changed their touch position and variance while still satisfying the required task, but we do not observe this trend. Together, the change in movement frequency, and the lack of change in task accuracy shows that robot as well as human co-workers influenced participant task performance in our experiment.

### Human form matters

Interestingly, covering the head and torso extinguished the contagions in the participant’s *htp* –the participant’s *htp*s were no longer affected in the robot covered co-worker (T(16) = -0.3, p = 0.78; dark blue data in Fig 6) and the human covered co-worker (T(12) = 0.24, p = 0.82; dark orange data in Fig 6), and these effects were significantly lower than the effects induced in the same participants in the robot co-worker condition (T(16) = 2.74, p = 0.028, Bonferroni corrected, one sample T-test between robot co-worker and robot covered co-worker; T(12) = 2.50, p = 0.054, Bonferroni corrected, one sample T-test between robot co-worker and human covered co-worker). This result show that the human form is crucial for induction of the performance changes.

In the robot co-worker and robot covered co-worker conditions, the robot played back the biological arm movements of a previous human volunteers. Finally, corresponding to previous studies that have shown that motor contagions are attenuated when a robot makes non-biological movements [9, 15], we added two control conditions (robot non-biol and robot indus) in which the participants could see the robot co-worker’s whole upper body, but the robot made non-biologically inspired arms movements to perform the task (see subsection HRP-2Kai movement trajectories). Consistent with previous studies we did not find any significant change in *htp*s in this condition (Z(16) = -1.07, p = 0.29 (cyan data); Z(17) = -0.28, p = 0.77 (magenta data) in Fig 6), and these values were different (tending to significance) compared to the robot co-worker condition of the same participants (robot non-biol condition: T(16) = 2.32, p = 0.066, Bonferroni corrected, one sample T-test) and (robot indus condition: T(17) = 2.53, p = 0.043, Bonferroni corrected, one sample T-test).

### The performance effect were implicit

An average of the scores from Q1, Q2, Q5 and Q4 (value subtracted from 7) was found to be equal to (mean±SD, 1.90±0.18) for the robot co-worker condition, and (mean±SD, 1.65±0.24) for the human co-worker condition respectively. These low scores suggested that the participants did not consciously realize the effects on their behavior. Q3 was used to confirm that the participants were not tired in our task. We obtained scores of (mean±SD, 0.96±0.18) across the participants in the robot co-worker condition, and (mean±SD, 0.75±0.22) in the human co-worker condition. Q6 was used to confirm that the participants did not hear any external audio cues from either the robot’s joints in the robot co-worker conditions (mean±SD, 0.5±1.25), nor the human co-worker’s touches in the human co-worker conditions (mean±SD, 0.58±1.36).

#### Contagion increases with robot exposure

**Fig 8:**
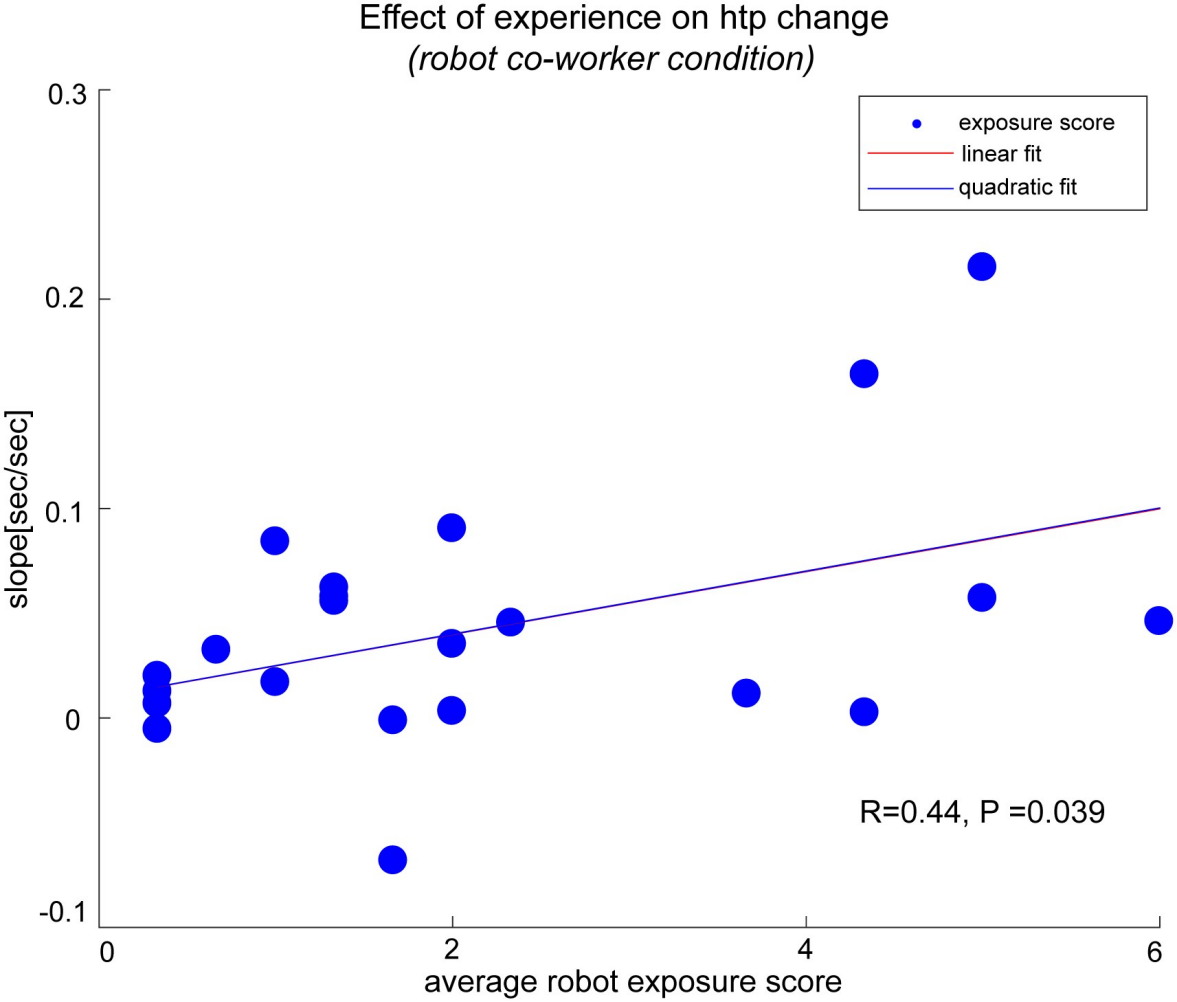
Robot experience exposure. The plot of the change of participant *htp*, with respect to their prior robot exposure and experience (self-scored by participants) showed a significant correlation between the two.

We received answers from 23 participants on our robot exposure questionnaire. Out of these participants, one participant who scored ‘0’ for all questions was removed. We averaged the scores (taking either one from RQ2 and RQ3, as they were complementary) for the others and plotted the average against their *htp* slope in the robot co-worker condition in Fig 8. Interestingly, we observed a significant positive correlation (Pearson’s R = 0.44, p = 0.039), such that the effect on the participants were larger if they had more exposure and experience with robots. This result is conform to a recent report where participants with more experience with robots show higher adaptation to it, see [31].

## Discussion

In summary, primarily, we observed that the performance frequencies of human participants were influenced by the presence of a humanoid robot co-worker (or a human co-worker). We observed that participants not only become slower with a slower co-worker, but also faster with faster co-workers. Here we were interested to see the change of participant behavior ‘relative’ to the robot behavior. Hence we looked at a ratio, hence to quantify the motor contagion, we analyzed change in participant’s *htp* between the together period and alone-period in a trial and relative to the *htp* of the co-worker behavior in the same trial. When the numerator term ($\tau_{p}^{t}\left(i\right)-\tau_{p}^{a}\left(i\right)$) is negative that means participants get faster from their initial participant-alone period *htp* (movement speed) and vice versa. Note that the subtraction in the denominator (of $Av(\tau_{p}^{a})$) is a constant that only shifts the curve and does not effect the slope.

In this study we wanted to choose a task that is simple, yet rich, and is representative of many industrial co-worker scenarios. We found that (repetitive) pick and place tasks to be the most common industrial tasks in which robots are employed. We therefore chose to start with a cyclic touch task in this experiment. The results we obtain here therefore, are specific to repetitive tasks. On the other hand, it has been shown that cyclic and discrete tasks may be very different in terms of neural processes [32], and further studies are required to verify whether the effects that we observe here are also valid for discrete movements. Further studies are also required to understand whether and how the contagions we observed here are related to *Motor entrainment*, which is a phenomena predominantly defined for rhythmic auditory stimuli [33, 34]. In our case we used white noise feedback to stop the participants from hearing noise from the moving robot. However, having said that, it is possible that the effect we observe here may be a form of *visual* Motor entrainment.

The performance frequencies of the participants were affected by the human and humanoid robot co-workers, their (press) task accuracy remained undisturbed (Fig 7). The effect on the human co-worker’s frequency and the absence of an effect on his accuracy, suggests that the performance of the human participants is affected by the presence of a robot co-worker; a slower robot co-worker reduces human performance (in terms of speed and accuracy), while a faster robot co-worker improves it. Previous studies have shown that specialized robots can influence both human performance and motivation during physical [35] and cognitive [36] interactions, our results here show that the mere presence of humanoid robots can induce effects in human performance.

Interestingly, the effect on the movement frequency was observed only when the head and torso of the co-worker was visible to the participants (Fig 6), indicating the crucial importance of the human form for these effects. Note that in order to investigate the effect of the visibility of the co-worker torso, we chose to cover the torso of the humanoid robot instead of using a different manipulator as a co-worker due to two main reasons. Primarily, this enabled us to create a condition where the physical appearance of the robot arm and its movement were identical between the robot co-worker and robot covered co-worker conditions. Furthermore, this helped us clarify that the contagions are not influenced by the *presence of a humanoid co-worker* (and the participant’s knowledge of it), but rather by the torso visibility. Both these issues would have been unclear with the use of a manipulator as a co-worker. On the other hand, our results open several new questions for future research perspectives. First, we observed that the visibility of the robot co-worker’s torso modulates contagions in a human co-worker, but the reasons behind this are still unclear. The effect is probably related to aspects of saliency as the torso not only occupies a larger visual field, but (especially the head and the eyes) also probably attracts participant attention when present. Second, in our task we examined the case where the robot co-worker made predominantly arm movements, while the torso remained static. While we believe the effect of the torso’s visibility should increase in tasks in which the torso moves, it remains to clarify how the torso movements affect contagions. Finally, while here we analyze a task where both co-workers (human and humanoid robot HRP-2Kai) and participants perform the same task, it would be interesting to analyze whether and how the contagions manifest in settings where the co-workers and participants work on different tasks, including non-industrial task that are explicitly collaborative, or competitive.

Quantitatively, the trends we observed were significant but not that substantial. However, within our participants, these trends were observed to increase with their participant’s robot experience (Fig 8), suggesting that they can prevail over a long time and are thus may important in scenarios involving long time robot-human interactions. Note that the questions used to quantify robot exposure represent the self perceived robot exposure by the participants rather than the actual robot exposure. A standard questionnaire to access actual robot exposure is absent and the development of one can be useful to understand how the effects, such as the one we highlight here, vary over time.

Overall, the results of our exploratory study highlight several new features of motor contagions, but also opens new questions for future research. These results can be useful for customizing the design of robot co-workers in industries and sports in order to moderate or exploit the contagions induced by them; contagions such as those related to body postures and undesirable competitions and that may affect worker health and psychology in long run, may be reduced by controlling the physical appearance and/or kinematics of robot co-workers, while where ethically valid, contagions may also be used to improve worker performance speed and hence productivity.

## Supporting information

S1 Video The video of our experiment explaining task protocol and data analysis is available here. https://goo.gl/9rqv4G.
(MP4)

S1 Fig All participants regression fits in the robot co-worker condition. Examples of linear regression fits obtained between the participant’s *htp* change between the together and alone conditions (ordinates), as a function of co-worker’s *htp*s (abscissa). Note that most participant plots show a positive slope indicating that the robot co-worker’s performance *htp* (hence frequency) influenced the human participants.
(TIF)

S2 Fig All participants regression fits in the human co-worker condition. Examples of linear regression fits obtained between the participant’s *htp* change between the together and alone conditions (ordinates), as a function of co-worker’s *htp*s (abscissa). The positive slopes show that the human co-worker’s performance *htp* (hence frequency) influenced the human participants.
(TIF)

S3 Fig All participants regression fits in the robot covered co-worker condition. Examples of linear regression fits obtained between the participant’s *htp* change between the together and alone conditions (ordinates), as a function of co-worker’s *htp*s (abscissa). Note that there is no trend in slopes across participant –the slopes were in fact observed to be zero across participants (Fig 6), indicating that the participant’s *htp*s were not affected in the robot covered co-worker condition.
(TIF)

S4 Fig All participants regression fits in the human covered co-worker condition. Examples of linear regression fits obtained between the participant’s *htp* change between the together and alone conditions (ordinates), as a function of co-worker’s *htp*s (abscissa). Like in S3 Fig, the slopes were observed to be zero across participants (Fig 6), indicating that the participant’s *htp*s were not affected in the human covered co-worker condition.
(TIF)

S5 Fig All participants regression fits in the robot non-biol co-worker condition. Examples of linear regression fits obtained between the participant’s *htp* change between the together and alone conditions (ordinates), as a function of co-worker’s *htp*s (abscissa). The plots again show that the participant’s *htp*s were not affected in the robot non-biol co-worker condition.
(TIF)

S6 Fig All participants regression fits in the robot indus co-worker condition. Examples of linear regression fits obtained between the participant’s *htp* change between the together and alone conditions (ordinates), as a function of co-worker’s *htp*s (abscissa). Like in S3 to S5 Figs, we observed no effect in the participants in the robot indus co-worker condition.
(TIF)
