# Supplementary figures and images for: More than just co-workers: Presence of humanoid robot co-worker influences human performance
Source: PLoS One. 2018 Nov 8;13(11):e0206698. doi: 10.1371/journal.pone.0206698 (PMC6224070; doi:10.1371/journal.pone.0206698)

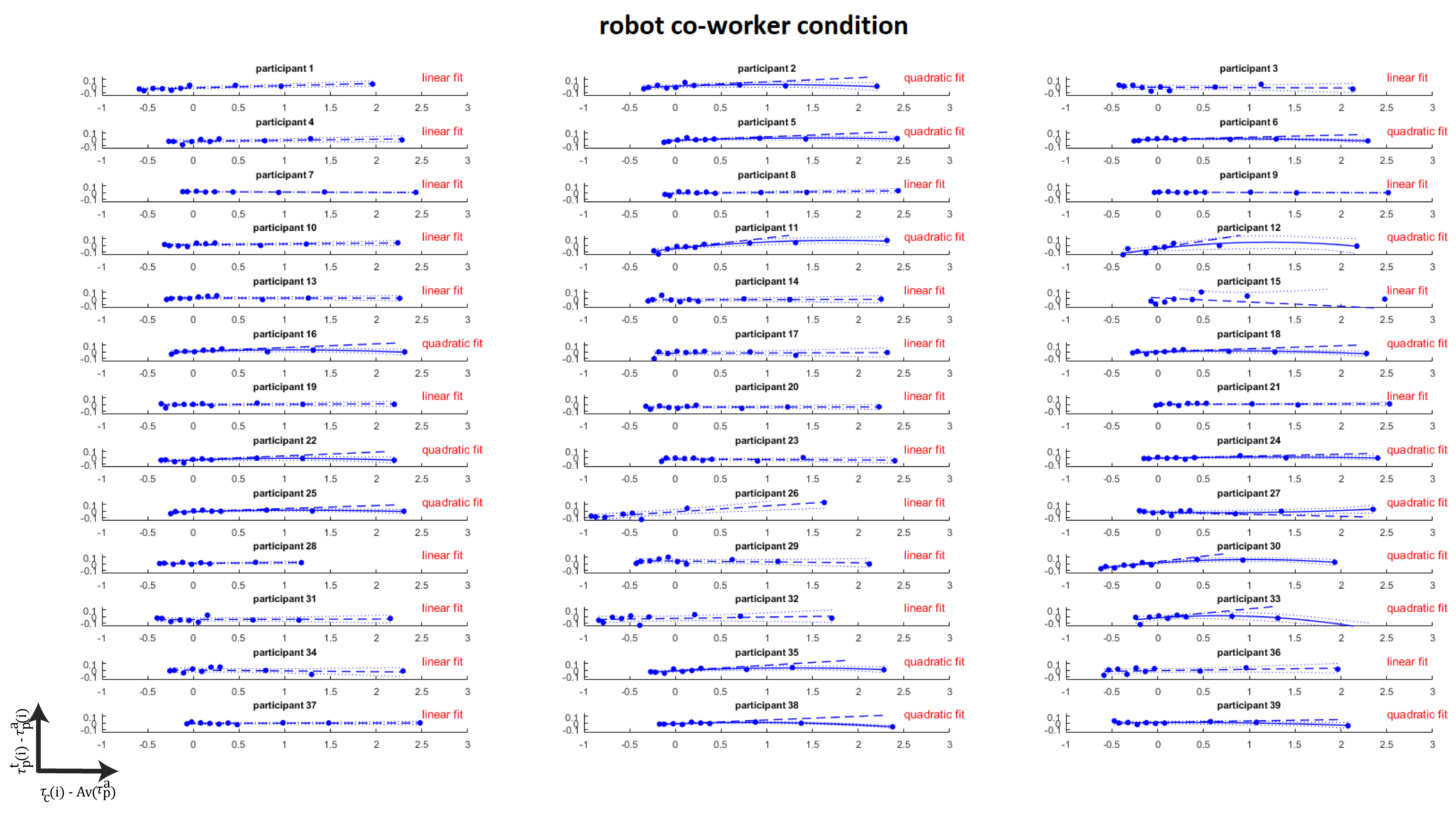

Supplement: S1 Fig — Examples of linear regression fits obtained between the participant’s htp change between the together and alone conditions (ordinates), as a function of co-worker’s htps (abscissa). Note that most participant plots show a positive slope indicating that the robot co-worker’s performance htp (hence frequency) influenced the human participants. (TIF) [file pone.0206698.s002.tif]

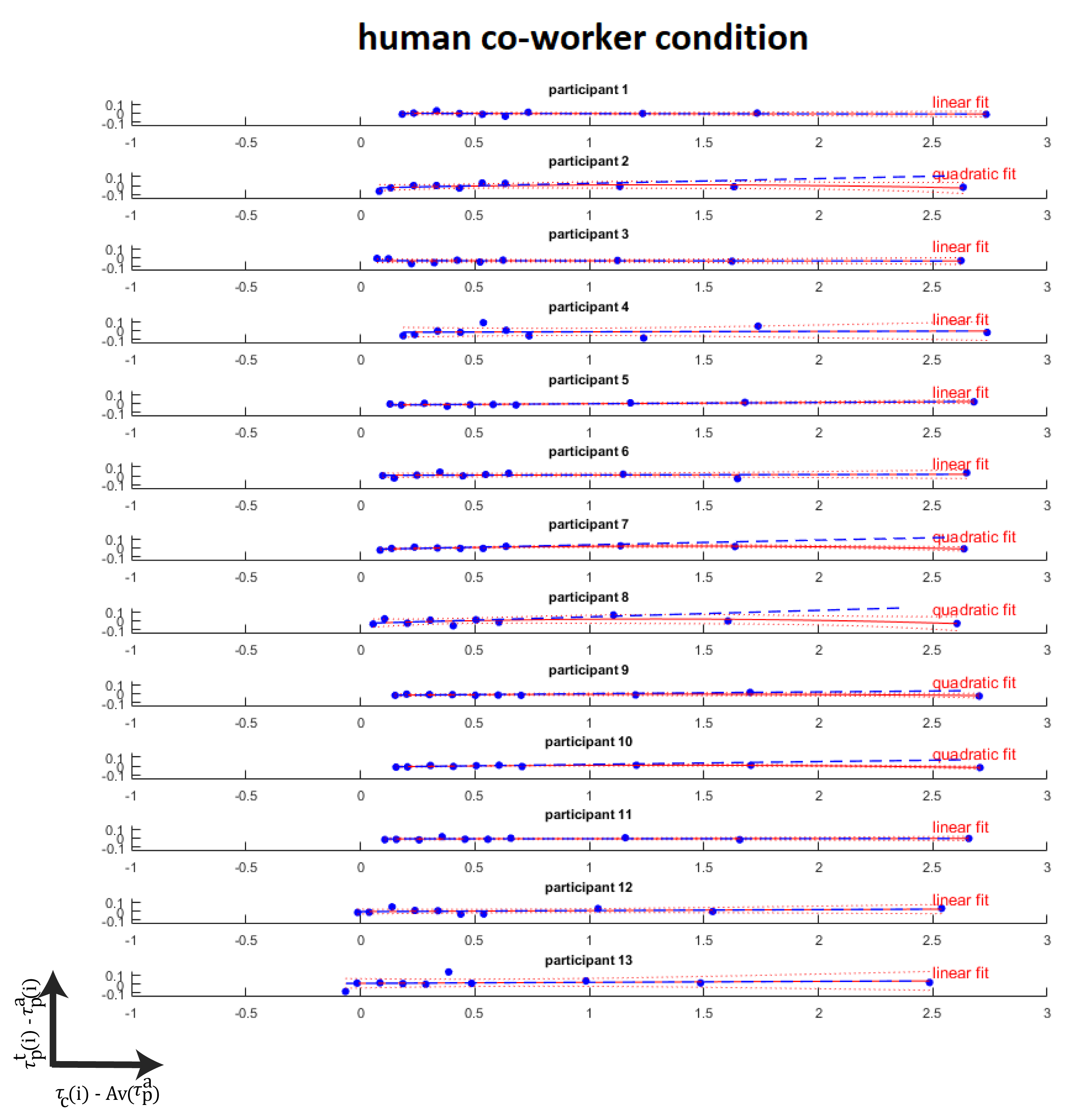

Supplement: S2 Fig — Examples of linear regression fits obtained between the participant’s htp change between the together and alone conditions (ordinates), as a function of co-worker’s htps (abscissa). The positive slopes show that the human co-worker’s performance htp (hence frequency) influenced the human participants. (TIF) [file pone.0206698.s003.tif]

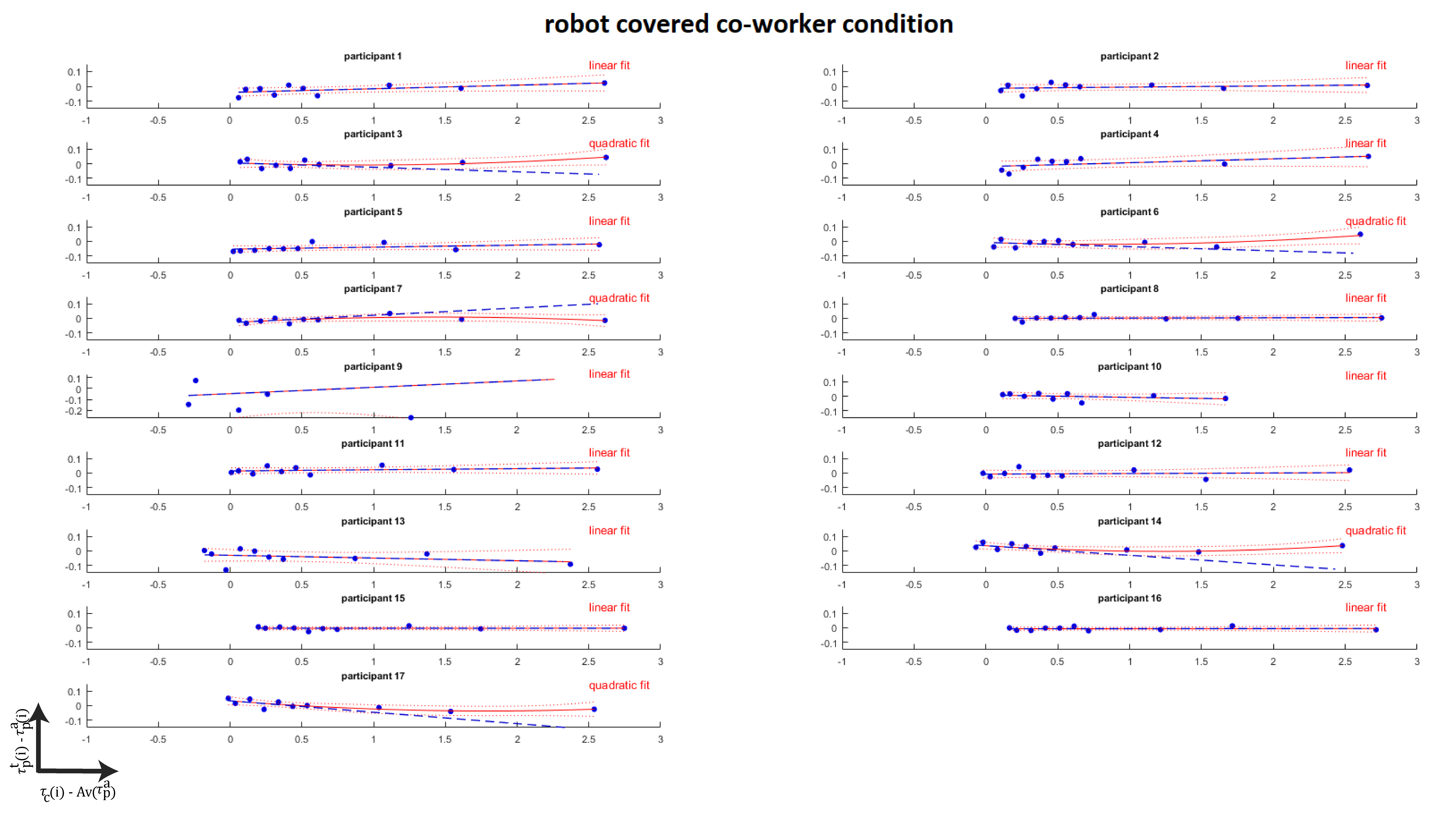

Supplement: S3 Fig — Examples of linear regression fits obtained between the participant’s htp change between the together and alone conditions (ordinates), as a function of co-worker’s htps (abscissa). Note that there is no trend in slopes across participant –the slopes were in fact observed to be zero across participants (Fig 6), indicating that the participant’s htps were not affected in the robot covered co-worker condition. (TIF) [file pone.0206698.s004.tif]

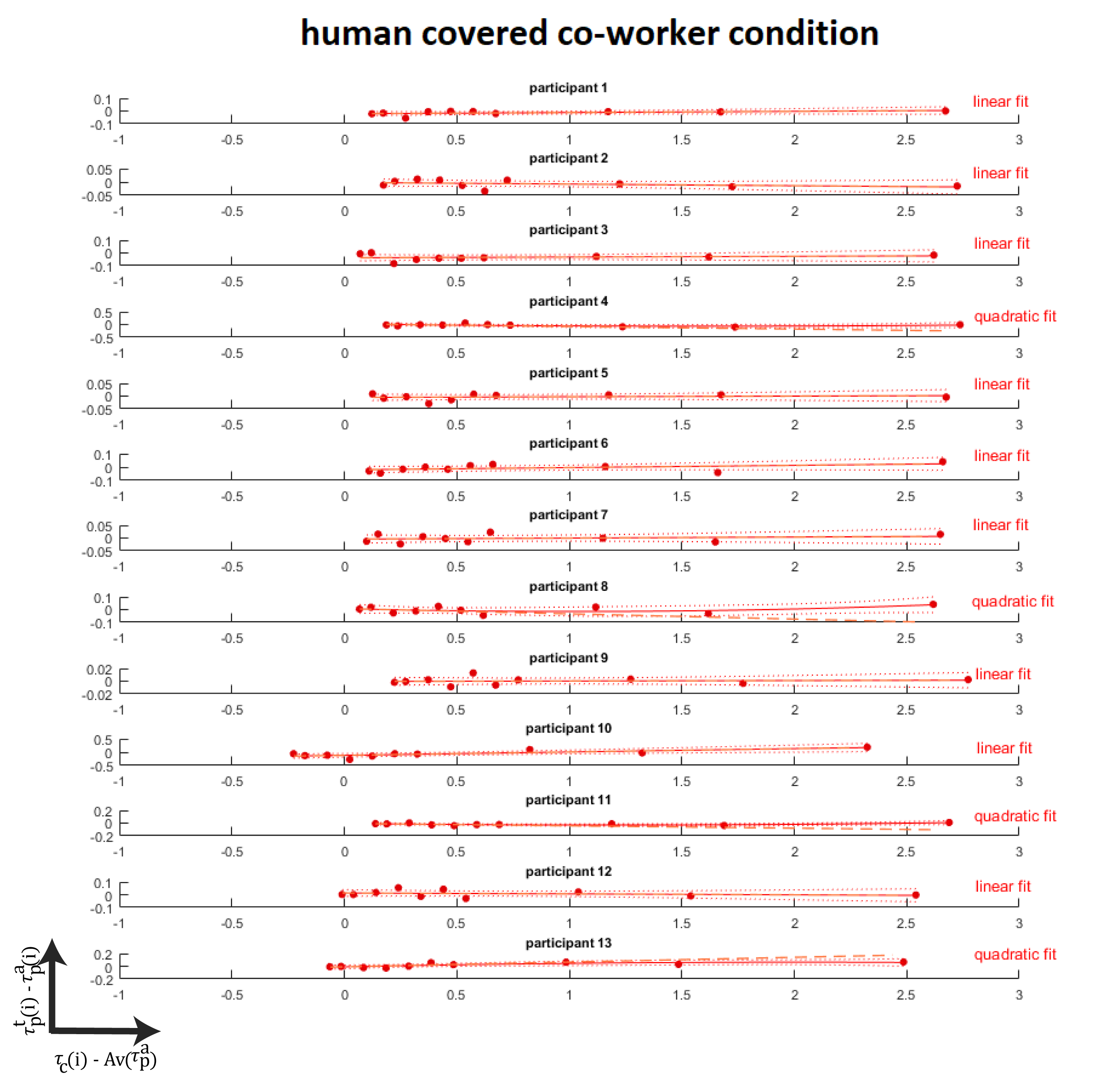

Supplement: S4 Fig — Examples of linear regression fits obtained between the participant’s htp change between the together and alone conditions (ordinates), as a function of co-worker’s htps (abscissa). Like in S3 Fig, the slopes were observed to be zero across participants (Fig 6), indicating that the participant’s htps were not affected in the human covered co-worker condition. (TIF) [file pone.0206698.s005.tif]

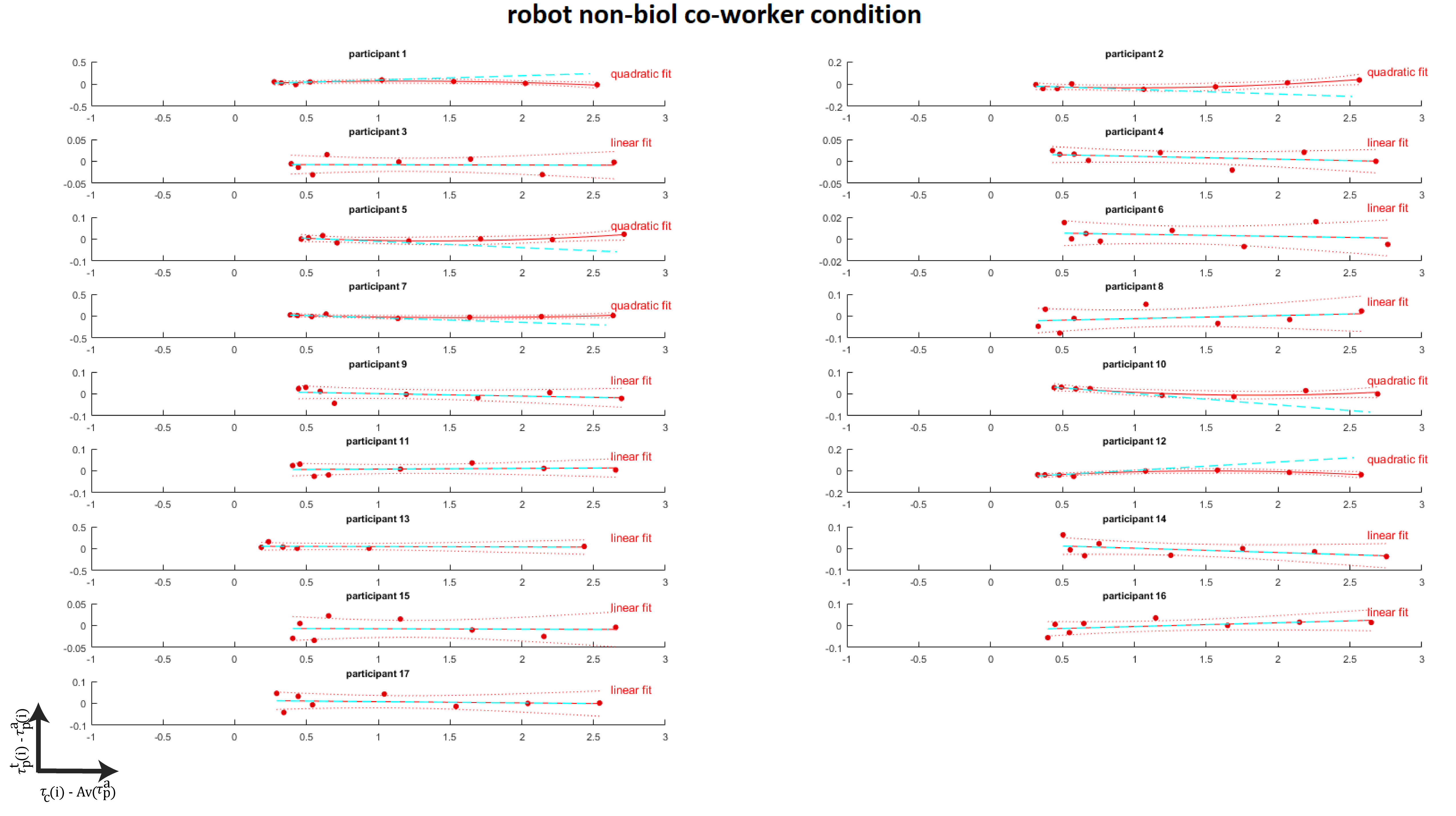

Supplement: S5 Fig — Examples of linear regression fits obtained between the participant’s htp change between the together and alone conditions (ordinates), as a function of co-worker’s htps (abscissa). The plots again show that the participant’s htps were not affected in the robot non-biol co-worker condition. (TIF) [file pone.0206698.s006.tif]

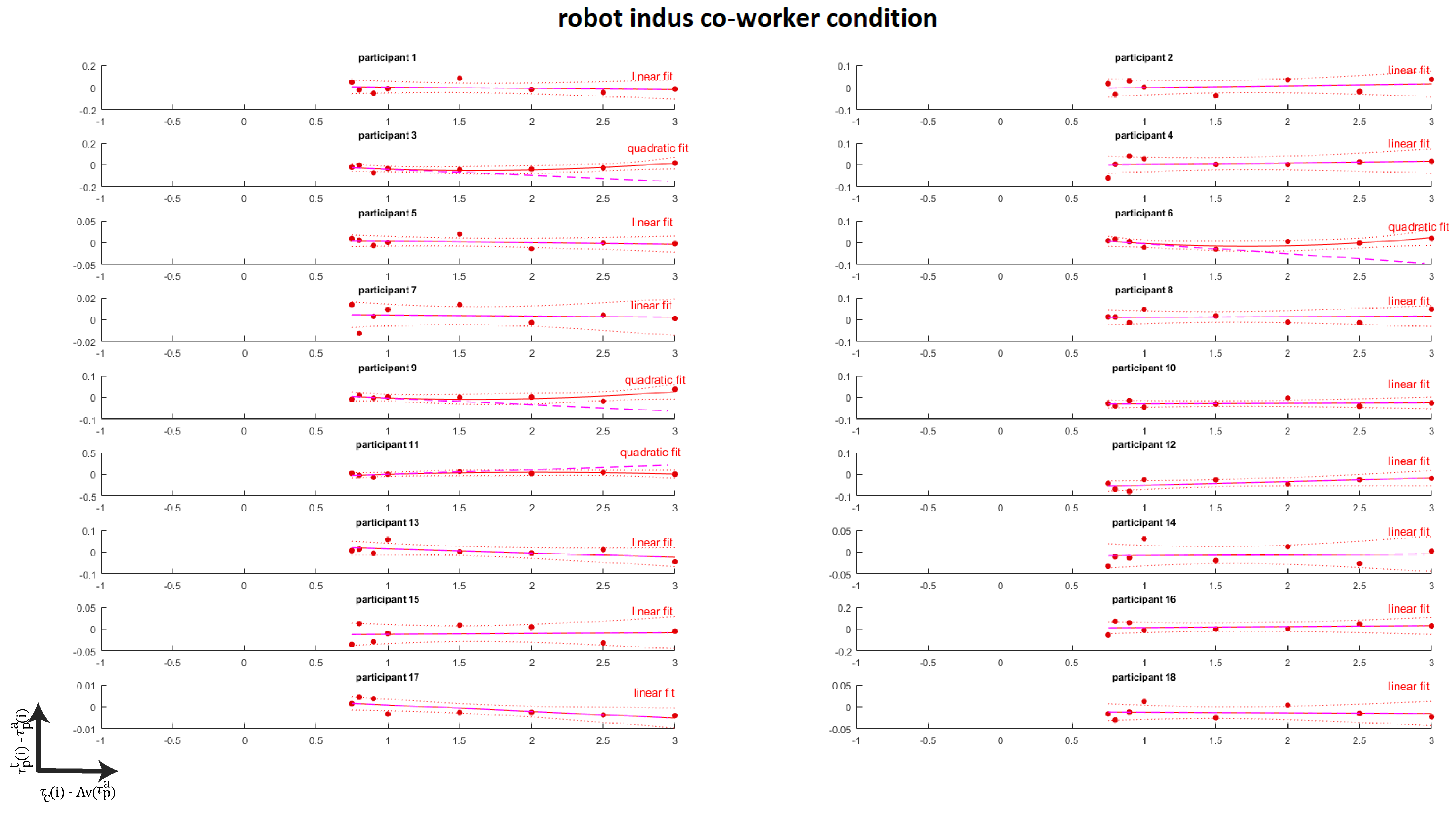

Supplement: S6 Fig — Examples of linear regression fits obtained between the participant’s htp change between the together and alone conditions (ordinates), as a function of co-worker’s htps (abscissa). Like in S3 to S5 Figs, we observed no effect in the participants in the robot indus co-worker condition. (TIF) [file pone.0206698.s007.tif]
